# A retrospective analysis of the link between the prognostic nutritional index and 1-year mortality rates in older adults suffering from femoral neck fractures

**DOI:** 10.3389/fnut.2026.1797064

**Published:** 2026-05-12

**Authors:** Pei-pei Li, Zi-ruo Zhang, Dan Chen, Lian Wen, Ru-yue Sun, Si-tong Yan, Jing Hu, Hong Zhi

**Affiliations:** 1 Comprehensive Ward, Honghui Hospital, Xi’an Jiaotong University, Xi’an, Shaanxi, China; 2 Foot and Ankle Orthopedic Diagnosis and Treatment Center, Honghui Hospital, Xi’an Jiaotong University, Xi’an, Shaanxi, China; 3 Department of Medicine, Yan’an University, Yan’an, Shaanxi, China; 4 Department of Internal Medicine, Honghui Hospital, Xi’an Jiaotong University, Xi’an, Shaanxi, China; 5 Department of Nursing, Honghui Hospital, Xi’an Jiaotong University, Xi’an, Shaanxi, China; 6 Department of Academic Development, Honghui Hospital, Xi’an Jiaotong University, Xi’an, Shaanxi, China

**Keywords:** elderly, femoral neck fracture, mortality, pronostic nutritional index, retrospective analysis

## Abstract

**Background:**

Femoral neck fractures are associated with high mortality rates in older adults, and nutritional status represents a potentially modifiable risk factor. The Prognostic Nutritional Index (PNI) serves as a simple, objective measure of nutritional and immune status; however, its capacity to predict 1-year mortality following femoral neck fractures surgery remains inadequately explored.

**Objective:**

This study aimed to: (1) examine the association between preoperative PNI and 1-year mortality after surgical treatment of femoral neck fractures in older adults, and (2) evaluate the predictive performance of PNI for postoperative survival.

**Methods:**

We conducted a retrospective cohort study at a tertiary academic medical center in China. Patients aged 60 years or older who underwent surgery for femoral neck fractures between January 2020 and January 2021 were included. Data on demographics, comorbidities, laboratory values (including serum albumin and lymphocyte count for PNI calculation), and 1-year mortality were extracted from electronic health records. Statistical analyses included multivariable logistic regression, restricted cubic spline (RCS) modeling, and receiver operating characteristic (ROC) curve analysis.

**Results:**

Among the 954 patients included in the analysis, 82 (8.6%) died within 1 year following surgery. Compared to survivors, patients in the mortality group had significantly lower preoperative prognostic nutritional index (PNI) scores (42.96 ± 4.64 vs. 45.92 ± 4.52, p < 0.001). In a multivariable-adjusted analysis accounting for age, sex, comorbidities, and type of surgery, a higher PNI remained independently associated with a reduced risk of 1-year mortality (OR = 0.94, 95% CI: 0.89–0.99, *p* = 0.025). A restricted cubic spline regression confirmed a continuous inverse association between PNI levels and mortality risk. The prognostic performance of PNI for predicting 1-year mortality was modest, with an area under the curve (AUC) of 0.68 (95% CI: 0.62–0.75). The optimal PNI cutoff value was 43.68, yielding a sensitivity of 71% and a specificity of 63%.

**Conclusion:**

Preoperative PNI is an independent predictor of 1-year mortality in older adults with femoral neck fractures. This simple and accessible marker may be incorporated into routine preoperative assessments to facilitate early identification of patients at heightened risk, supporting targeted nutritional interventions in perioperative nursing care.

## Introduction

Femoral neck fracture (FNF) accounts for 53% of all proximal femoral fractures and pose a significant risk to the elderly population (1). As the aging demographic grows, the incidence rate of FNF is on the rise (2). The World Health Organization projects that hip fractures linked to osteoporosis will increase threefold in the next 50 years, escalating from 1.7 million cases in 1990 to 6.3 million by 2050 globally (3). This type of injury leads to considerable morbidity and mortality among older adults, with 1-year mortality rates ranging from 25 to 29% in various studies (4, 5). A meta-analysis encompassing 142 studies (1,139,752 patients across 14 countries/regions) found that 1-year mortality rates varied, with Singapore reporting 10.8% [95% CI 9.6–12.1], while Australia and New Zealand had rates of 23.3% [95% CI 22.3–24.5] and 23.8%, respectively (5). A systematic review indicated that the pooled 1-year mortality rate was 17.47% following femoral intertrochanteric fractures and 9.83% after femoral neck fractures from 2000 to 2018 (6). Postoperative complications and mortality rates were heightened in hip fracture patients exhibiting signs of malnutrition, significantly impacting both their short-term and long-term quality of life (7). Surgical procedures in older individuals provoke an inflammatory and metabolic stress response, leading to increased catabolism and muscle deterioration, which is particularly harmful for frail elderly patients with diminished muscle mass (8). Due to the often subtle and overlooked nature of malnutrition in this age group, regular nutritional assessments are crucial. Research has shown that malnutrition considerably affects postoperative results across diverse patient populations, with prevalence rates between 20 and 50% among hospitalized individuals (9, 10). Dubé et al. (11) noted malnutrition rates reaching 50% in patients undergoing joint replacement surgeries. The prognostic nutritional index (PNI), first introduced by Buzby et al. (12) and later refined by Onodera et al. (13), serves as a straightforward method for assessing perioperative nutrition by analyzing preoperative serum albumin (Alb) and total lymphocyte count (TLC). Previous studies suggest that preoperative PNI may be a reliable indicator of long-term survival for surgical cancer patients (14, 15), offering good predictive accuracy, objective metrics, and ease of use. As a simple and feasible nutritional detection index, PNI is widely used to evaluate the occurrence of perioperative complications and predict the prognosis (16, 17). PNI is calculated from two routine, standardized preoperative blood tests. This makes it a highly practical, objective, and low-cost screening tool, which is particularly advantageous for frail, elderly patients who may be unable or unwilling to participate in more complex, questionnaire-based nutritional assessments. However, it remains uncertain whether the PNI can effectively forecast the long-term outcomes for patients in this demographic. This study aimed to examine the association between preoperative PNI and 1-year mortality after surgical treatment of femoral neck fractures in older adults and evaluate the predictive performance of PNI for postoperative survival. Early identification of high-risk patients allows for targeted interventions, which may enhance survival rates and improve recovery outcomes.

## Materials and methods

### Participants and data source

Between January and December of 2021, a retrospective observational research project took place at Honghui Hospital, which is part of Xi’an Jiaotong University in China. The research adhered to the guidelines set forth in the Declaration of Helsinki and received ethical clearance from the Ethics Committee of the Faculty of Medicine at Honghui Hospital (No. 202403060). Patient information, including both clinical and biochemical data, was carefully gathered from electronic health records. To qualify for inclusion, participants had to meet specific criteria: the study subjects included patients aged ≥ 60 years (18) with a history of femoral neck fracture, as well as those who underwent surgical treatment for femoral neck fracture, including Total Hip Arthroplasty (THA), partial hip replacement, and internal fixation. Femoral neck fractures were distinguished from other types of hip fractures based on preoperative anteroposterior and lateral hip radiographs. Fractures were classified as femoral neck if the fracture line was proximal to the basicervical region. Patients with intertrochanteric or subtrochanteric fractures were excluded from this analysis. In cases where radiographic evaluation was ambiguous, computed tomography (CT) scans were used for confirmation.

Exclusions were made for cases that: (1) involved fractures in various areas, or (2) stemmed from high-energy trauma, (3) involved individuals requiring revision surgery, or (4) had medical records that were incomplete or lacked sufficient data, impacting the comprehensive assessment of the case, specifically if more than 10% of the data was missing.

In 2021, Honghui Hospital at Xi’an Jiaotong University in China conducted a retrospective observational study, which received approval from the Ethics Committee. This study involved the systematic collection of comprehensive patient data, encompassing demographic information, pre-existing health issues, the nature of surgical procedures, anesthesia methods used, intraoperative blood transfusion details, American Society of Anesthesiologists (ASA) scores, and the duration of hospital stays. For biochemical assessments, data on hemoglobin, white blood cell counts, red blood cell counts, platelet levels, lymphocyte counts, urea, creatinine, and serum albumin were documented upon patient admission. The study also calculated the Hemoglobin Platelet Ratio (HPR), Platelet to Lymphocyte Ratio (PLR), and the Prognostic Nutritional Index (PNI).

PNI was determined from the last laboratory tests performed prior to surgery, during the period from admission to the operative day. In cases with multiple preoperative evaluations, the final measurement before surgery was recorded. The PNI evaluates nutritional and immune status using serum albumin and total lymphocyte counts, calculated as PNI = serum albumin (g/L) + 5 × lymphocytes (× 10^9^/L) (19).

The primary outcome assessed was the 1-year survival rate of elderly patients who underwent surgery for femoral neck fractures. One-year survival status was determined through structured telephone follow-ups conducted by trained research staff who were blinded to the patients’ baseline PNI levels. A standardized questionnaire was used to confirm patient vital status. For patients who could not be reached directly, we contacted their next of kin. Mortality was confirmed by the date and, when available, cause of death as reported by family members. The follow-up was complete for 954 of the 1,057 eligible patients, and this rate is now explicitly stated.

A total of 1,622 patients were enrolled, eliminated 349 patients younger than 60, 54 who did not receive surgical intervention, 5 with high-energy injuries (like those from vehicle collisions or falls from significant heights), 48 with fractures in other locations, 2 who requiring reoperation, and 59 who had insufficient information on over three variables. Furthermore, 48 patients had incomplete data regarding hemoglobin, platelets, serum albumin, or lymphocyte counts, while 103 were untraceable for follow-up. Consequently, the analysis ultimately comprised data from 954 patients. Of the 1,057 eligible patients, 103 (9.7%) were lost to follow-up and were excluded from the final analysis. We did not perform imputation due to the observational nature of the study and the relatively low proportion of incomplete cases.

### Statistical analysis

Continuous variables were summarized using means and standard deviations (SD) or medians along with interquartile ranges (IQR). To compare groups, either a *t*-test or Mann-Whitney U-test was utilized. Count data were represented as percentages (%), and inter-group comparisons were conducted using the χ^2^-test or Fisher’s exact test. Multivariable logistic regression analysis was performed to identify factors independently associated with 1-year mortality. Logistic regression was chosen because the primary outcome was binary, and exact survival times were not available in our dataset, precluding the use of time-to-event methods such as Cox proportional hazards models. Restricted cubic spline (RCS) regression was employed to flexibly model the relationship between PNI (as a continuous variable) and the log odds of 1-year mortality, allowing for potential non-linearity. The number of knots was set at four, located at the 5th, 35th, 65th, and 95th percentiles of the PNI distribution. To formally test for non-linearity, a likelihood ratio test was performed comparing the model with the linear PNI term to the model including the spline terms. A significant *p*-value for the spline terms would indicate a non-linear relationship. The results are presented graphically, showing the estimated OR (with 95% CI) across the range of PNI values, with the reference value set at the median PNI. Additionally, a receiver operating characteristic (ROC) curve was generated to assess the predictive capability of PNI for 1-year mortality, with the optimal cutoff value identified via the Youden index. Statistical analyses were performed using R version 4.3.3, with a significance threshold set at *p* < 0.05 (two-tailed).

## Results

### Demographics

Out of 954 participants, 872 (91.4%) were classified in the Survival group, while 82 (8.6%) were in the Mortality group. The Mortality group had a higher average age (mean age 84.39 ± 5.77 years compared to 74.63 ± 8.57 years; *p* < 0.001) and underwent shorter surgeries [mean duration 90.00 min (IQR 70.00–115.25) versus 80.00 min (IQR 60.00–100.00); *p* = 0.006). The median hospital stay was 7.00 days (IQR 6.00–9.00) overall, 7.00 days (IQR 6.00–9.00) in the Survival group, and 8.00 days (IQR 6.00–10.00) in the Mortality group (*p* = 0.030). BMI (*p* = 0.066) did not show significant differences. Additionally, the Mortality group had a greater percentage of males (41.46% compared to 28.33%; *p* = 0.013) and a higher incidence of chronic conditions (82.93% versus 65.71%; *p* = 0.002). The ASA classification varied significantly between the two groups (*p* < 0.001), with a larger share of ASA 2 in the Mortality group (62.96% versus 31.72%). There were no notable differences in anesthesia type (p = 0.177) or intraoperative blood transfusions (p = 0.058). However, the types of surgeries differed significantly (p < 0.001), with the Mortality group primarily undergoing Partial-hip replacement procedures (97.56% compared to 63.19%) (see Table 1).

**TABLE 1:** Baseline clinical characteristics of elderly femoral neck fracture patients (*n* = 954).

Variables	Total (*n* = 954)	Survival (*n = 872*)	Mortality (n = 82)	Statistic	*P*
Age (years), mean (SD)	75.47 ± 8.80	74.63 ± 8.57	84.39 ± 5.77	*t* = –13.95	< 0.001
Operation duration (min), M (Q_1_, Q_3_)	90.00 (68.25, 115.00)	90.00 (70.00, 115.25)	80.00 (60.00, 100.00)	*Z* = –2.76	0.006
LOS(d), M (Q_1_, Q_3_)	7.00 (6.00, 9.00)	7.00 (6.00, 9.00)	8.00 (6.00, 10.00)	*Z* = –2.17	0.030
BMI (kg/m^2^), Mean ± SD	21.69 ± 7.03	21.82 ± 7.31	20.32 ± 2.24	*t* = 1.84	0.066
Gender, n(%)				χ^2^ = 6.23	0.013
Female	673 (70.55)	625 (71.67)	48 (58.54)		
Male	281 (29.45)	247 (28.33)	34 (41.46)		
Concomitant chronic diseases, n(%)				χ^2^ = 10.08	0.002
NO	313 (32.81)	299 (34.29)	14 (17.07)		
YES	641 (67.19)	573 (65.71)	68 (82.93)		
ASA score, n(%)				χ^2^ = 34.99	< 0.001
1	594 (62.46)	568 (65.29)	26 (32.10)		
2	327 (34.38)	276 (31.72)	51 (62.96)		
3	30 (3.15)	26 (2.99)	4 (4.94)		
Type of anesthesia, n (%)				–	0.177
GA	945 (99.06)	865 (99.20)	80 (97.56)		
RA	9 (0.94)	7 (0.80)	2 (2.44)		
Intraoperative blood transfusion, n(%)				χ^2^ = 3.60	0.058
No	783 (82.08)	722 (82.80)	61 (74.39)		
Yes	171 (17.92)	150 (17.20)	21 (25.61)		
Surgery type, (%)				χ^2^ = 39.57	< 0.001
THA	276 (28.93)	274 (31.42)	2 (2.44)		
Partial-hip replacement	631 (66.14)	551 (63.19)	80 (97.56)		
Internal fixation	47 (4.93)	47 (5.39)	0 (0.00)		

*t*, *t*-test, χ^2^, Chi-square test, -, Fisher exact; SD, standard deviation; M, Median, Q_1_, 1st Quartile, Q_3_, 3st Quartile; Z, Mann-Whitney test; LOS, Length of stay; BMI, Body mass index; GA, general anesthesia; RA, regional anesthesia; THA, Total Hip Arthroplasty.

In a study involving 954 individuals, the Mortality group exhibited reduced red blood cell counts (average 3.84 ± 0.68 compared to 4.00 ± 0.55; *p* = 0.013) and lower hemoglobin concentrations (116.74 ± 15.12 versus 121.88 ± 15.71; *p* = 0.005). Additionally, the PNI scores were diminished in the Mortality group (42.96 ± 4.64 against 45.92 ± 4.52; *p* < 0.001). This group also had elevated creatinine levels [64.00 (IQR 52.25–83.50) versus 59.00 (IQR 50.00–70.00); *p* = 0.004] and increased urea levels [6.65 (IQR 5.40–8.57) compared to 5.50 (IQR 4.50–7.00); *p* < 0.001]. However, no significant differences were found in white blood cell counts (*p* = 0.910), platelet counts (*p* = 0.636), HPR (*p* = 0.523), PLR (*p* = 0.200), or lymphocyte counts (*p* = 0.080) between the two groups (see Table 2).

**TABLE 2:** Biochemical characteristics of elderly femoral neck fracture patients (*n* = 954).

Variables	Total (*n* = 954)	Survival (*n* = 872)	Mortality (*n* = 82)	Statistic	*P*
WBC(× 10^9^/L), Mean ± SD	7.49 ± 2.49	7.49 ± 2.49	7.52 ± 2.42	*t* = –0.11	0.910
RBC(× 10^12^/L), Mean ± SD	3.99 ± 0.56	4.00 ± 0.55	3.84 ± 0.68	t = 2.48	0.013
PLT(× 10^9^/L), Mean ± SD	186.83 ± 70.62	186.43 ± 68.88	191.15 ± 87.40	*t* = –0.48	0.636
HB(g/L), Mean ± SD	121.44 ± 15.72	121.88 ± 15.71	116.74 ± 15.12	t = 2.84	0.005
HPR, M (Q_1_, Q_3_)	0.70 (0.53, 0.89)	0.70 (0.54, 0.89)	0.65 (0.47, 0.91)	*Z* = –0.64	0.523
PLR, M (Q_1_, Q_3_)	162.00 (119.48, 217.86)	164.29 (120.20, 218.51)	156.67 (107.12, 204.84)	*Z* = –1.28	0.200
LYMPH (× 10^9^/L), M (Q_1_, Q_3_)	1.08 (0.81, 1.46)	1.09 (0.81, 1.47)	1.01 (0.77, 1.34)	*Z* = –1.75	0.080
PNI, Mean ± SD	45.66 ± 4.61	45.92 ± 4.52	42.96 ± 4.64	t = 5.64	< 0.001
Creatinine (μmol/L), M (Q_1_, Q_3_)	59.00 (51.00, 71.00)	59.00 (50.00, 70.00)	64.00 (52.25, 83.50)	*Z* = –2.86	0.004
BUN (mmol/L), Mean ± SD	5.60 (4.60, 7.10)	5.50 (4.50, 7.00)	6.65 (5.40, 8.57)	*Z* = –3.86	< 0.001

*t*, *t*-test; SD, standard deviation; Z, Mann-Whitney test; M, Median, Q_1_, 1st Quartile, Q_3_, 3st Quartile; PLT, Platelet; HB, hemoglobin; WBC, white blood cell; RBC, red blood cell; HPR, hemoglobin platelet ratio; PLR, Platelet to lymphocyte ratio; LYMPH, lymphocyte; PNI, prognostic nutritional index; BUN, blood urea nitrogen. Laboratory values were obtained from the last preoperative assessment prior to surgery. PNI was calculated using preoperative serum albumin and lymphocyte counts.

### Subgroup analysis

As detailed in Table 3, PNI was significantly associated with reduced mortality across most subgroups, including sex and comorbidity categories. However, the results for certain subgroups should be interpreted with caution due to limited sample sizes and few outcome events. In the ASA III subgroup (*n* = 30, with only 4 deaths), the odds ratio was 0.79 (95% CI: 0.58–1.08, *P* = 0.134), which did not reach statistical significance. This null finding may reflect insufficient statistical power rather than a true lack of association. Similarly, for patients undergoing internal fixation (*n* = 47), no deaths occurred in this subgroup, rendering the odds ratio non-calculable and precluding any meaningful inference. These exploratory findings highlight the need for further validation in larger, adequately powered studies.

**TABLE 3:** Subgroup analysis of the association between PNI and mortality of elderly femoral neck fracture patients.

Variables	n (%)	OR (95%CI)	*P*	P for interaction
All patients	954 (100.00)	0.87 (0.83 ∼ 0.92)	< 0.001	
Gender				0.828
Female	673 (70.55)	0.87 (0.82 ∼ 0.93)	< 0.001	
Male	281 (29.45)	0.88 (0.82 ∼ 0.95)	0.002	
Concomitant chronic diseases				0.747
No	313 (32.81)	0.89 (0.80 ∼ 0.99)	0.031	
Yes	641 (67.19)	0.87 (0.82 ∼ 0.92)	< 0.001	
ASA score				0.159
1	594 (62.46)	0.84 (0.77 ∼ 0.92)	< 0.001	
2	327 (34.38)	0.93 (0.87 ∼ 0.99)	0.022	
3	30 (3.15)	0.79 (0.58 ∼ 1.08)	0.134	
Surgery type				0.813
THA	276 (28.93)	1.01 (0.71 ∼ 1.43)	0.959	
Partial-hip replacement	631 (66.14)	0.90 (0.86 ∼ 0.95)	< 0.001	
Internal fixation	47 (4.93)	1.00 (non-calculable)	1.000	

OR, odds ratio; CI, confidence interval.

### Mortality

**FIGURE 1:**
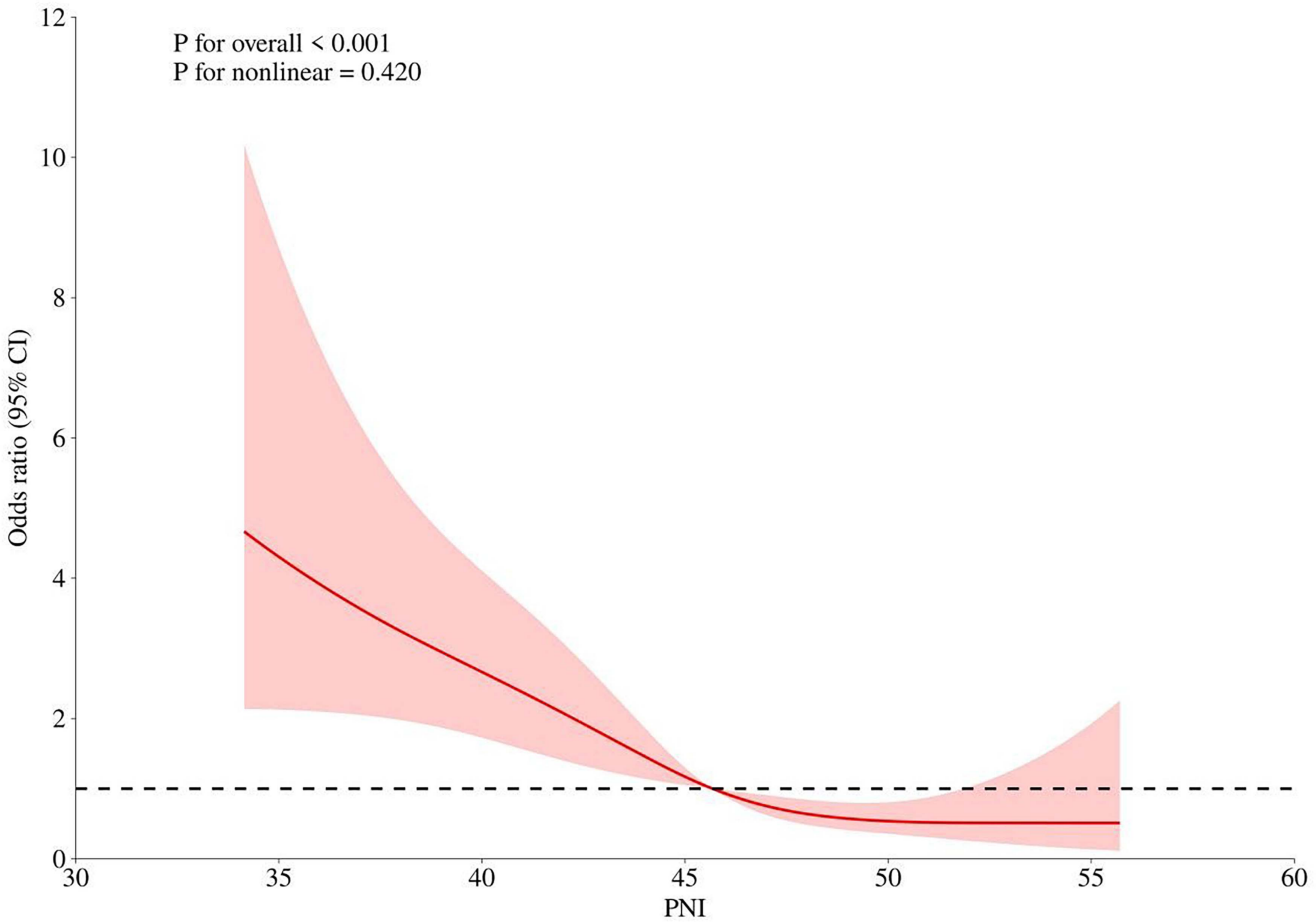
Association between admission PNI and 1-year all-cause mortality using restricted cubic spline (RCS) models.

According to the multivariate analysis shown in Table 4, being male raised the mortality risk by a factor of 1.85 (*p* = 0.020). Additionally, for every year of age, the mortality risk rose by 1.14 (*p* < 0.001) on its own. Conversely, the PNI variable was linked to a lower mortality rate (OR = 0.94, *p* = 0.025). The presence of chronic illnesses and the type of surgery did not correlate with an increased likelihood of death. Furthermore, the RCS models indicated that a rise in admission PNI corresponded with a steady reduction in the risk of mortality (Figure 1).

**TABLE 4:** Multivariate analysis of mortality in elderly femoral neck fracture patients.

Variables	β	S.E	*Z*	*P*	OR (95%CI)
Intercept	–11.70	2.35	–4.97	< 0.001	0.00 (0.00 ∼ 0.00)
Gender					
Female					Reference
Male	0.61	0.26	2.33	0.020	1.85 (1.10 ∼ 3.09)
Concomitant chronic diseases					
No					Reference
Yes	0.59	0.32	1.81	0.070	1.80 (0.95 ∼ 3.40)
Surgery type					
THA					Reference
Partial-hip replacement	1.42	0.75	1.89	0.058	4.14 (0.95 ∼ 18.00)
Internal fixation	–12.96	881.88	–0.01	0.988	0.00 (non-calculable)
Age	0.13	0.02	6.19	< 0.001	1.14 (1.09 ∼ 1.19)
PNI	–0.07	0.03	–2.25	0.025	0.94 (0.88 ∼ 0.99)

OR, odds ratio; CI, confidence interval.

### The significance of PNI in forecasting the risk of death after surgery in older individuals with fractures of the femoral neck

**FIGURE 2:**
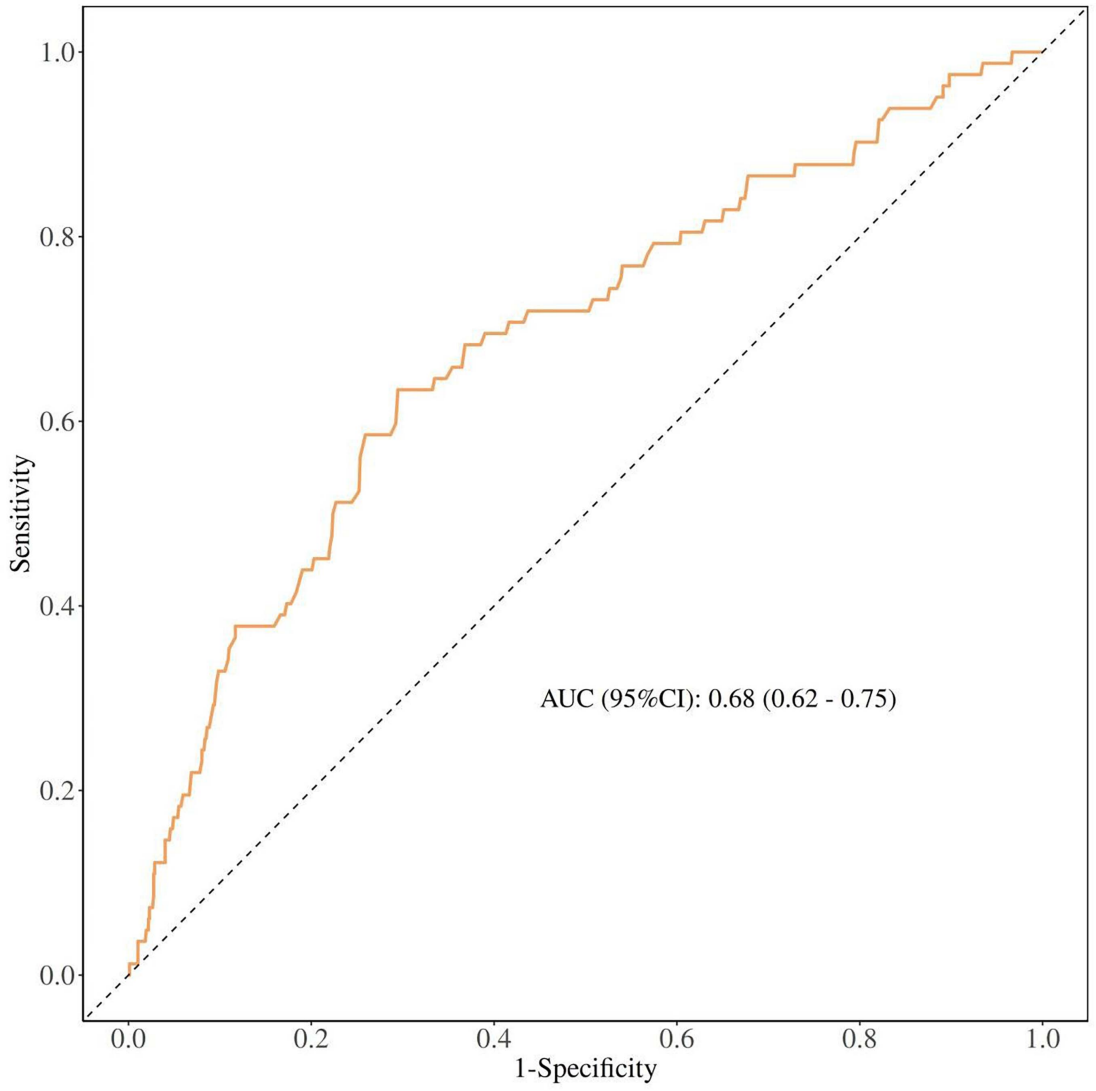
ROC curve of PNI for predicting postoperative mortality in elderly patients with femoral neck fractures.

The predictive significance of PNI for mortality in older patients with femoral neck fractures was assessed quantitatively. The area under the receiver operating characteristic curve (AUC) measured 0.68 (95% CI: 0.62–0.75, *p* < 0.001), reflecting a moderate level of discrimination. At the ideal cutoff point of 43.68, sensitivity reached 0.71 while specificity was recorded at 0.63 (see Figure 2 and Table 5).

**TABLE 5:** Clinical value of PNI in predicting postoperative mortality in elderly patients with femoral neck fractures.

AUC (95%CI)	Sensitivity	Specificity	Cut off
0.68 (0.62–0.75)	0.71	0.63	43.68

## Discussion

The research provides compelling proof that the preoperative PNI is a dependable indicator of 1-year overall mortality in older adults undergoing surgery for femoral fractures. Those with elevated PNI scores exhibited a markedly reduced likelihood of dying within a year. A PNI threshold of 43.68 was determined to be the most effective for forecasting 1-year mortality. These results suggest that PNI levels may be useful in pinpointing elderly patients who are at an increased risk of adverse outcomes after femoral fracture surgery.

In a study of 263 hip fracture patients aged ≥ 70 years, malnourished individuals exhibited significantly higher long-term mortality than their well-nourished counterparts (38.5% vs. 18.9%, p < 0.05). Multivariable analysis confirmed malnutrition as an independent risk factor for mortality (HR 0.269; 95% CI 0.085–0.859; *p* < 0.05) (20). This finding aligns with a larger prospective cohort study (*n* = 942) that identified the Prognostic Nutritional Index (PNI) as an independent predictor of long-term survival in older adults with hip fractures, reporting an association between higher PNI scores and reduced 3-year mortality (21). The present study extends these findings by demonstrating a dose-response relationship, whereby each unit increase in PNI incrementally lowered mortality risk. This association remained robust after adjustment for potential confounders. Although receiver operating characteristic (ROC) analysis identified an optimal binary cutoff of 43.68 for clinical risk stratification, restricted cubic spline (RCS) analysis revealed that the inverse relationship between PNI and mortality is continuous. This finding suggests that PNI is best interpreted as a continuous marker, with progressively lower values indicating escalating risk a nuance that complements the dichotomous approach. The protective effect was most pronounced within the PNI range of 35–45, clinically validating the ROC-derived cutoff. Regarding predictive performance, PNI demonstrated moderate accuracy in predicting postoperative mortality in our cohort of femoral neck fracture patients (AUC: 0.68 [0.62–0.75], *p* < 0.001). An AUC of 0.68 indicates moderate predictive ability, suggesting that PNI alone is not sufficient as a standalone screening tool for identifying high-risk patients. Rather, PNI should be integrated into a comprehensive risk assessment that includes other clinical factors such as age, comorbidities, ASA classification, and surgical type. This finding is consistent with existing literature, although some variability exists. For instance, a large-scale study reported similar AUCs ranging from 0.64 to 0.68 for predicting mortality at various postoperative time points, corroborating our finding of moderate predictive strength (7). The study demonstrated that preoperative PNI can predict postoperative ICU admission and in-hospital mortality in elderly hip fracture patients. A PNI < 32.5 was identified as the optimal cutoff for mortality prediction, with an AUC of 0.660 (95% CI: 0.516–0.803) (22). Conversely, He et al. reported a higher AUC of 0.772 in a similar cohort, suggesting marginally superior predictive ability (23). However, their study did not address the clinical implications of the low baseline PNI values (37.83 in patients without complications and 37.05 in those with complications), which independently indicate substantial malnutrition. Wang et al. stratified patients into risk categories based on PNI quartiles (≤ 43.23, 43.23–47.35, > 47.35) and found that higher categories were associated with fewer complications and lower 2-year mortality (24). While these thresholds are consistent with our ROC-derived cutoff of 43.68, their study did not establish an optimal binary cutoff, potentially limiting direct clinical application. In contrast, Shirakabe et al. investigated PNI in the context of acute heart failure, establishing lower cutoffs that underscore the importance of disease-specific interpretation (25). The PNI offers practical advantages: it is derived from routine assessments of lymphocyte and albumin levels, ensuring objectivity and minimal patient burden, which renders it particularly suitable for older adults, including those with cognitive impairments. While its predictive value is consistent with studies on other nutritional indices such as the CONUT score or GNRI, a direct comparison was beyond the scope of this study. The prognostic significance of PNI likely reflects the combined impact of nutritional deficiency and immune compromise on postoperative outcomes (26, 27); however, the molecular mechanisms linking the Prognostic Nutritional Index (PNI) to clinical outcomes remain unclear. As a quantitative marker encompassing both nutritional and immune status, PNI-associated malnutrition and immune compromise are likely key contributors to poor prognosis in patients with femoral neck fractures (24).

Several aspects of the subgroup analyses require clarification. Table 3 shows a consistent inverse association between PNI and mortality in most strata. However, caution is needed for certain subgroups because of small sample sizes and few outcome events. In the ASA III subgroup, comprising only 30 patients and 4 deaths, the odds ratio had a wide confidence interval and did not reach statistical significance. This finding likely reflects insufficient statistical power, representing a false-negative result rather than a true absence of predictive value in this population. In the Internal fixation subgroup, all 47 patients survived the 1-year follow-up. With zero deaths, the odds ratio could not be calculated, making the result clinically uninformative for mortality prediction. These data remain in the table for transparency and to show the full scope of the analysis. These findings highlight the need for large, multicenter, prospective studies with sufficient statistical power to confirm the prognostic value of PNI across different surgical types and risk groups.

### Study strengths and limitations

The research involved 954 older adults suffering from femoral neck fractures, and the high rate of follow-up contributed to a robust sample size, which is crucial for a precise evaluation of the link between PNI and mortality after surgery. Nonetheless, the study has several limitations. First, as a retrospective, single-center analysis, the findings may not be generalizable to other populations or settings; multicenter studies are needed to validate our results. Second, although mortality data were collected prospectively via telephone follow-up, 103 patients (9.7%) were lost to follow-up and excluded from the analysis, which may introduce selection bias. Third, due to the retrospective design, we were unable to adjust for several important clinical factors known to influence femoral neck fractures mortality, including frailty indices, timing of surgery, perioperative complications, and postoperative rehabilitation protocols. The absence of these variables represents a potential source of residual confounding. Fourth, the subgroup analyses, particularly for ASA III patients (*n* = 30, with only 4 deaths) and the internal fixation group (*n* = 47, with no deaths), were limited by small sample sizes and few outcome events. Consequently, these estimates are unstable and should be interpreted as exploratory. The lack of statistical significance in the ASA III subgroup may reflect insufficient statistical power (type II error) rather than a true absence of association, and the internal fixation group provided no evaluable data on mortality. Fifth, because our follow-up only recorded vital status at 1 year without documenting the exact date of death, we were unable to perform time-to-event analyses, which are generally preferred for mortality outcomes. Although logistic regression is appropriate for a binary endpoint, it does not account for the timing of events and may be less efficient than survival analysis. Future studies should collect precise survival times to enable more robust analytical approaches. Lastly, we have added a strong statement emphasizing that the optimal cutoff value of 43.68 was derived from a single-center retrospective cohort and requires external validation in independent, prospective, and ideally multi-center studies before it can be broadly recommended for clinical practice.

## Conclusion

In summary, Preoperative PNI is a promising predictor of 1-year mortality risk in elderly patients with femoral neck fractures. Its cutoff value of 43.68 needs further validation in larger-scale, multicenter prospective studies.

## Data Availability

The data supporting the findings of this study are available from the corresponding author upon reasonable request.
